# Psychological well-being and needs of parents and carers of children and young people with mental health difficulties: a quantitative systematic review with meta-analyses

**DOI:** 10.1136/bmjment-2023-300971

**Published:** 2024-08-02

**Authors:** Faith Martin, Dania Dahmash, Sarah Wicker, Sarah-Lou Glover, Charlie Duncan, Andrea Anastassiou, Lucy Docherty, Sarah Halligan

**Affiliations:** 1 School of Psychology, Cardiff University, Cardiff, UK; 2 Coventry University, Coventry, UK; 3 Parental Minds, Honiton, UK; 4 British Association for Counselling and Psychotherapy, Lutterworth, UK; 5 University of Bath, Bath, UK

**Keywords:** data interpretation, statistical, adult psychiatry, child & adolescent psychiatry

## Abstract

**ABSTRACT:**

**Question:**

For parents of children and young people (CYP) with diagnosed mental health difficulties, what are the levels of parents’ well-being and psychological need?

**Study selection and analysis:**

Medline, PsycINFO, EMBASE, AMED, CINAHL, Web of Science and Cochrane Library of Registered Trials were searched from inception to June 2023. Inclusion criteria: parents of CYP aged 5–18 years with formal mental health diagnosis. Data were extracted from validated measures of well-being or psychological needs with established cut-off points or from a controlled study.

**Findings:**

32 of the 73 310 records screened were included. Pooled means showed clinical range scores for one measure of depression, and all included measures of anxiety, parenting stress and general stress. Meta-analyses showed greater depression (g=0.24, 95% CI 0.11 to 0.38) and parenting stress (g=0.34, 95% CI 0.20 to 0.49) in parents of CYP with mental health difficulties versus those without. Mothers reported greater depression (g=0.42, 95% CI 0.18 to 0.66) and anxiety (g=0.73, 95% CI 0.27 to 1.18) than fathers. Narrative synthesis found no clear patterns in relation to CYP condition. Rates of parents with clinically relevant levels of distress varied. Typically, anxiety, parenting stress and general stress scored above clinical threshold. Quality appraisal revealed few studies with a clearly defined control group, or attempts to control for important variables such as parent gender.

**Conclusions:**

The somewhat mixed results suggest clinical anxiety, parenting and general stress may be common, with sometimes high depression. Assessment and support for parents of CYP with mental health problems is required. Further controlled studies, with consideration of pre-existing parental mental health difficulties are required.

**PROSPERO registration number:**

CRD42022344453.

**WHAT IS ALREADY KNOWN ON THIS TOPIC**

- Mental health difficulties are common among children and young people (CYP).
- There are known, complex bi-directional links between parent and child mental health; however, there has been no synthesis of quantitative research examining the psychological well-being and needs specifically of parents of CYP with mental health difficulties.

**WHAT THIS STUDY ADDS**

- Our findings suggest depression, anxiety, parenting stress and general stress may be high in parents of CYP with mental health difficulties, with greater depression and parenting stress compared with parents of CYP without mental health difficulties, and greater distress in mothers compared with fathers of CYP with mental health difficulties.
- The state of the current research makes clear recommendations for future studies.

**HOW THIS STUDY MIGHT AFFECT RESEARCH, PRACTICE OR POLICY**

- This study highlights the need for more case-control studies to address this issue, and for studies that attempt to measure distress in parents specific to their CYP’s mental health difficulty.
- Clinicians should be mindful of the likelihood of parents’ difficulties and consider interventions to support them.

## Background

 Many mental health difficulties, including anxiety disorders, obsessive compulsive disorder (OCD), eating disorders and mood disorders, have their onset in childhood and youth, many before age 18 years.^1 2^ Approximately one in five young people (aged 5–18 years) in Europe have a diagnosed mental health difficulty.^3^ Importantly, many of these young people live with their families or with an adult caring for them, who may be impacted by the young person’s distress. Often there are long waiting lists for assessment and diagnosis,^4^ and limited access to clinical intervention^5^ and therefore families may be managing the situation without professional support.

There is significant evidence linking pre-existing parental mental health difficulties and parenting behaviours with CYP mental health outcomes, highlighting bi-directional links.^6^,^8^ A review focusing on reported prevalence of mental illness in parents of CYP receiving mental health treatment, found rates of between 16% and 79% of parental mental illness, highlighting the importance of attention to parental well-being.^9^ However, that review focused on prevalence only, and did not address wider issues of parenting or general stress, nor an examination of data for parents of CYP with and without mental health difficulties. Parents influence CYP mental health, for example, through parent-child interaction^7^ and parenting practices such as discipline and communication.^10 11^ CYP mental health can influence parents’ well-being owing to the distress of seeing their child struggle.^12^ This can then influence the family environment and parenting behaviours, creating a cycle of interactions.^13^ Thus, a crucial step in understanding how families can be supported with CYP mental health problems requires identifying the extent of distress in parents who may need support themselves. Furthermore, supporting parents may improve the parents’ well-being, and improve CYP outcomes through better communication with their CYP, a better family environment and increased parental modelling of coping strategies.

Potential consequence of CYP mental health difficulties for parental well-being have been documented across multiple problems and domains. Parents of CYP who self-harm described significant distress.^14^ Qualitative studies have shown the impact on parents’ lives and well-being, linked to their CYP’s mental health.^15^ In relation to CYP anxiety and depression, qualitative evidence highlighted parental feelings of guilt, helplessness and sadness, and needing to hiding their own support needs.^16 17^ Parental experiences in relation to attention deficit hyperactivity disorder (ADHD) have been particularly well-studied, with existing systematic reviews highlighting that parents of CYP with ADHD had relatively higher rates of mental health difficulties,^18^ higher levels of parenting stress^19^ and worse quality of life,^20^ as well as elevated levels of depression specifically in mothers.^21^

The well-being of parents of CYP with other mental health difficulties is less well-documented and detailed synthesis is lacking. Given that mothers tend of be most engaged in CYP service use, and an observed tendency to place greater responsibility or even blame on mothers,^22^ it may be that the impact differs by parent type. Mothers’ continue to typically be responsible for the majority of childcare and report high levels of responsibility and self-blame for CYP mental health.^23 24^ However, the majority of participants in studies with parents of CYP with mental health difficulties are mothers, meaning it remains important to examine the well-being of fathers also. A comprehensive picture of parental well-being in the context of wider CYP mental disorders is currently lacking, which limits the extent to which potential parental support needs are being acknowledged and met.

### Objective

This review aimed to synthesise the quantitative studies that measure current well-being and psychological needs in parents of CYP with mental health difficulties. The objectives were to investigate reported levels of well-being, compare well-being between parents of child with and without a mental health diagnosis, comparing mothers and fathers of CYP with mental health difficulties, to highlight gaps in knowledge about these parents’ well-being and to examine potential need for intervention to support these parents.

### Study selection and analysis

A protocol was registered (PROSPERO CRD42022344453) and published.^25^ Following changes were made: separation of reporting of qualitative and quantitative needs/well-being studies into two manuscripts; focus on parents of CYP with mental health conditions other than ADHD, owing to existing ADHD-focused reviews^19 21^; removal of searches in Open Grey, Social Policy and Practice and Applied Social Sciences Index, as scoping found few relevant records via these sources and inclusion of meta-analyses.

### Search methods for identification of studies

Medline, PsycINFO, EMBASE, AMED, CINAHL, Web of Science (complete core collection) and Cochrane Library of Registered Trials were searched, covering all records from database inception until 23 June 2023. English language limits were applied at search stage. The search was composed of four blocks, with terms for (1) parents, (2) children and young people, (3) mental health diagnostic terms and (4) psychological state, impact/experiences/needs (full search strategies in online supplemental materials 2).

### Study selection

The titles and abstracts of all identified studies were downloaded and managed in Rayyan software.^26^ Duplicates were removed automatically and checked manually. Titles and abstracts were screened independently by at least two reviewers, with discrepancies resolved by a third. Full-texts were accessed (or requested) for all studies that were included at this stage and were also assessed independently by two reviewers, and discrepancies resolved by a third reviewer if necessary.

### Inclusion and exclusion criteria

Inclusion criteria were:

1. Adults in a parent/carer role (biological, step-parents, relatives assuming parenting role, non-biological and adoptive parents, foster carers and other adults with a legal guardian role).
2. Parents had a CYP aged 5–18 years (at least 50% of sample), aligning with many Child and Adolescent Mental Health Services (CAMHS) in the UK, Australia and many European services.^27^
3. CYP diagnosed by an appropriate professional with a mental health condition, for example, depression disorders, anxiety disorders, OCD, oppositional defiant behaviour (), conduct disorder, internalising and externalising disorders, eating disorders, bipolar or psychoses and emerging personality disorders.
4. Provided quantitative data relating to ‘current’ (rather than lifetime or historic) parents’ well-being and needs: including but not limited to knowledge, parents’ mental health, parenting satisfaction, family relationships, parenting self-efficacy.
5. Used validated measure that *either* has established cut-off scores *or* was used with a control group of parents of CYP without mental health difficulties, to allow interpretation of the results.

Exclusion criteria were:

1. Qualitative studies; reviews.
2. Studies focused on post-traumatic stress disorder (PTSD), given potential overlap with parents’ own experiences of any shared traumatic events and existing reviews of relationships of CYP PTSD to parental psychological health.^28^
3. Studies focused on parents of CYP with special educational needs, including autism spectrum conditions or developmental language disorders, owing to likely additional needs for these parents, existing reviews^29 30^ and our focus specifically on CYP with mental health conditions.
4. Studies not in English.

While we included studies focused on ADHD in our search, due to a very large number of studies all studies with parents of CYP with ADHD are excluded from this report and will be reported separately (in preparation).

### Data extraction

Data were extracted into an Excel spreadsheet by one reviewer and checked by another for accuracy. Data relating to study location, design, CYP age, CYP diagnosis, sample sizes, parent gender, measurement tools used and results on included measures were extracted. Mean scores with SD were extracted where possible, to allow calculation of pooled means where appropriate. Where not reported, counts of number of parents reaching clinical levels of distress, median scores or other summary scores as reported were extracted. To enable meta-analyses, results were extracted separately, where reported, for case versus control groups (parents of children without mental health difficulties), and for mothers versus fathers.

Where an intervention was reported, only baseline data were extracted as the focus of this review is on overall well-being and needs, rather than intervention effectiveness. Baseline scores of intervention or control groups were pooled where necessary. All relevant data were extracted.

### Data analysis

Pooled means, pooled SD and 95% CIs of the pooled means were calculated, where the same measure had been used in at least three studies overall. These were then compared with published measurement cut-off points, to indicate if mean scores were within clinical range. To facilitate this, a table of cut-off scores for all included measures was created (online supplemental materials 3).

For studies that included either (a) a control group (ie, parents of CYP without any mental health condition), allowing comparison between case-control parents or (b) provided data separately for mothers and fathers of CYP with mental health difficulties to allow comparison, meta-analysis was conducted where k≥3^31^ provided usable data on the same variable. No prior meta-analyses results were included, as no relevant prior analyses were identified. Reported data were used to calculate standardised mean differences (SMDs). This was done for continuous data, and dichotomous data, to allow combination in analysis with continuous data.^32^,^34^ SMDs were calculated using RevMan^35^ using reported N, SD for continuous data. For reported dichomotomous data (eg, count of parents reaching clinical depression vs not), OR and SE were calculated using RevMan, converted to SMD using a standard formula (SMD=(√3/π)lnOR and SE=(√3/π)).^32 34^ A random effects model was used in all cases (using RevMan V.5.4^35^), owing to heterogeneity (measures and samples). To assess heterogeneity, τ^2^ and I^2^ were calculated, with cut-offs of 0%, 25%, 50% and 75% applied to indicate no, low, moderate or considerable heterogeneity, respectively.^36^ Effect size Hedge’s g is reported.^37^ For analyses including dichotomous data (alone or in combination), a generic inverse variance approach random effects model generated pooled effect sizes. Sensitivity analysis was conducted by excluding one study at a time.

A narrative synthesis was also conducted.^38^ We also synthesised findings from repeated measures/longitudinal studies that provide data (not related to intervention) over time to understand changes in parental state.

### Quality appraisal

We conducted quality appraisal using an adapted Newcastle Ottawa Scale,^39^ covering issues around participant selection, comparability and outcomes. The adaptations are detailed in online supplemental materials 4, with the results.

### Findings

### Study characteristics

**Figure 1:**
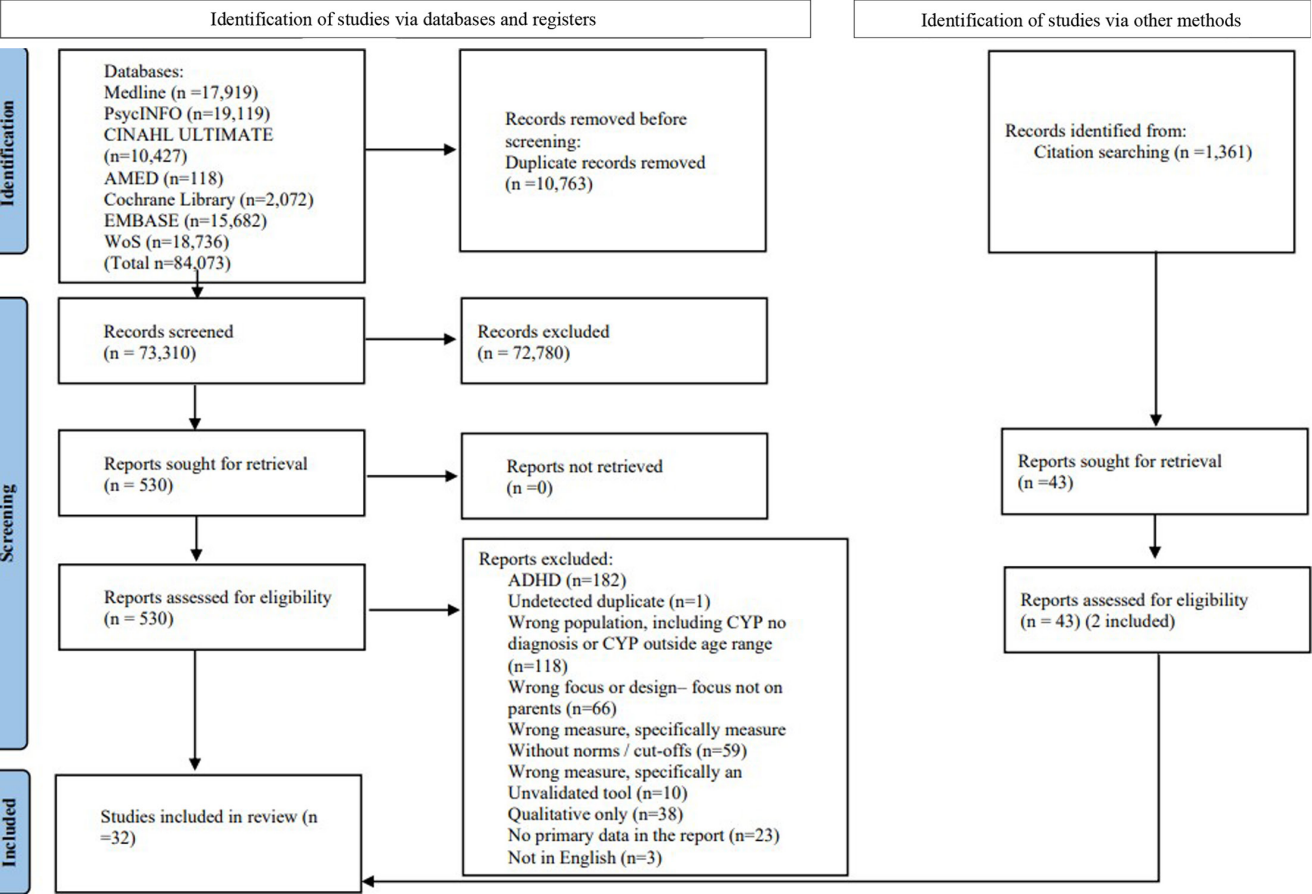
Identification of studies via databases and registers.

A total of 32 studies were included in the review. Figure 1 provides Preferred Reporting Items for Systematic Reviews and Meta-Analyses flow chart.

The study characteristics are summarised in online supplemental materials 1. Measures used covered depression, anxiety, parenting stress, general stress, overall mental health, work and social adjustment and coping. Several excluded studies included measures of parenting self-efficacy. However, due to the absence of a control group and established cut-offs, it was not possible to interpret these scores, and therefore they were excluded.

The majority of studies were conducted in Northern Europe (k=10), the USA (k=8), Asia (k=7) and Australia (k=5), with an additional two from Southern Europe and Middle East (k=2). No studies were from South America or Africa.

The studies included parents of CYP with mood disorders (total k=15): anxiety (k=7), anxiety and OCD (k=1), anxiety and depression (k=2), depression alone (k=3), depression and bipolar (k=1) and bipolar alone (k=1). Several included CYP with externalising difficulties (k=8). Eating disorders were also represented (k=7). The remaining two covered any mental health diagnosis and psychoses.

Quality appraisal (online supplemental materials 4) found that 14/32 studies (44%) had scores indicating poor quality (using cut-off less than five stars).^40^ This also highlighted a lack of studies that include a clearly defined, well-selected control group, together with an overall poor level of quality in relation to comparability and control for confounding factors, such as parent’s gender.

### Pooled means

Table 1 reports pooled means and indicates whether each measure is in the clinical range. Depression predominantly did not fall within the clinical range, whereas anxiety, parenting stress and general stress typically had pooled means in the clinical range.

**Table 1:** Pooled means of parents’ depression, anxiety, parenting stress and general stress, presented by measure

Variable	Measure	k	N	Pooled mean (pooled SD)	95% CI	Pooled mean in clinical range?	Measure cut-off scores for reference
Depression	BDI-II	6	882	11.1 (8.05)	10.6 to 11.6	No	0–13 minimal 14–19 mild 20–28 moderate 29–63 severe
Depression	BDI-II	Anorexia only 4	779	11.4 (8.03)	10.8 to 12	No	As above
Depression	DASS—as DASS-42	5	1283	15.1 (16.23)	14.2 to 16	Moderate	10–13 mild 14–20 moderate 21–27 severe +28 extremely severe
Depression	CES-D	3	781	13.6 (9.11)	13 to 14.2	No	16+ clinically significant
Anxiety	STAI state	4	430	43.7 (11.0)	42.7 to 44.7	Yes	>40
Anxiety	STAI trait	3	398	41.0 (10.01)	40 to 42	Yes	>40
Anxiety	DASS—as DASS-42	4	1283	9.0 (12.01)	8.34 to 9.66	Mild	4–5 mild 6–7 moderate 8–9 severe 10+ extremely severe
Parenting stress	PSI-SF total	4	509	106.6 (21.67)	105 to 108	Yes	>90 clinical stress
General stress	DASS—as DASS-42	3	752	28.4 (18.47)	27.1 to 29.7	Severe	15–18 mild 19–25 moderate 26–33 severe 34+ extremely severe

### Meta-analyses

A total of five studies were case-control^41^,^45^ and nine studies^4245^,^52^ provided data from mothers and fathers separately, although one did not provide sample size details, so was excluded from synthesis.^46^ Four meta-analyses were conducted: two case-control comparisons (depression, parenting stress) and two mother-father comparisons (depression and anxiety). Forest plots are provided in online supplemental materials 5.

#### Case-control comparisons

For depression scores (where dichotomous and continuous outcomes were combined), the overall combined difference was statistically significant (k=4, n=1518 (case 623, control 895), g=0.24, 95% CI 0.11 to 0.38, p=0.0003), with a small effect size. Parents of children with mental health problems had worse (higher) depression scores than parents of children without mental health problems. Heterogeneity was low (I^2^=13%), with effect sizes all favouring control. Results must be interpreted with caution owing to the small k.

Comparing case-control scores for parent stress scores (where all data were continuous), the overall combined difference was statistically significant (k=3, n=1302 (case 487, control 815), g=0.34, 95% CI 0.20 to 0.49, p<0.00001), with a small effect size. Parents of children with mental health problems had worse (higher) parenting stress scores than parents of children without mental health problems. Heterogeneity was low (I^2^=27%). Effect sizes all favoured control. Results must be interpreted with caution owing to the small k.

#### Comparing scores from mothers-fathers

Comparing mothers’ and fathers’ scores for depression scores (where dichotomous and continuous outcomes were combined), the overall combined difference was statistically significant (k=7, n=3552 (mothers 1818, fathers 1734), g=0.42, 95% CI 0.18 to 0.66, p<0.003), with a small effect size. Mothers had worse depression (higher scores) than fathers. Heterogeneity was low (I^2^=27%), with effects all in the same direction. Although k=7, this remains a small number of studies, and together with the large 95% CIs and several SMDs that cross 0, these results must be interpreted with caution.

Comparing mothers’ and fathers’ scores for anxiety scores (where dichotomous and continuous outcomes were combined), the overall combined difference was statistically significant (k=3, n=334 (mothers 197, fathers 137), g=0.73, 95% CI 0.27 to 0.1.18, p<0.002), with a medium-large effect size. Mothers had worse anxiety (higher scores) than fathers. Heterogeneity was moderate-considerable (I^2^=70%), although all effects were in the same direction. However, with a small number of included effect sizes based on data from 334 participants in total, high heterogeneity and a 95% CI for 1 SMD that crosses 0, results are tentative.

#### Sensitivity analyses

The results did not persist following sensitivity analysis for two comparisons (online supplemental materials 6). For case-control depression, removing He *et al*^44^ led to greater weight for the two studies where 95% CI of SMD crossed zero.^41 45^ For mother-father anxiety, removing Duclos *et al*^48^ led to greater weight similarly to a study where 95% CI of SMD crossed zero.^42^

### Narrative synthesis

#### Clinical levels of distress

Table 2 summarises if mean scores were within clinical range by study, in relation to variable, CYP condition and measure used. This highlights variation in levels of non-clinical/clinical distress reported.

**Table 2:** Summary of parents’ mean scores in relation to clinical threshold for each measure

Measure used	Child diagnosis	Results below threshold	Results above threshold
Parental depression			
BDI	Anxiety or depression*	Tan and Rey (2005)^45^ Racey *et al *(2018)^63^	–
	Eating disorder (any)	Schwarte *et al* (2019)^50^ Truttman *et al *(2020)^64^ Duclos *et al* (2023)^48^	Zeiler *et al* (2023)^65^
CES–D	Externalising†	Hamovitch *et al *(2019)^10^ He *et al* (2020)^44^ He *et al* (2021)^66^	Gerkensmeyer *et al* (2008)^67^
DASS	Anxiety or depression	Johnco *et al* (2021)^11^	Poole *et al* (2018)^68^ Halldorsson *et al* (2018)^46^
	Eating disorder (any)		Wilksch (2023)^55^
Other	Anxiety or depression	Alqahtani and Osman (2021)^58^	Fields (2012)^54^
	Eating disorder (any)	Stewart *et al* (2017)^69^	
	Externalising		Lim and Shim (2021)^49^
	Other diagnoses	–	Algorta *et al* (2018)^41^ Sengupata *et al* (2017)^51^
Parental anxiety			
DASS	Anxiety or depression	Johnco *et al *(2021)^11^	Poole *et al *(2018)^68^ Halldorrsson *et al* (2018)^46^ ‡
	Eating disorder (any)	–	Wilksch (2023)^55^
STAI	Eating disorder (any)	–	Truttman *et al *(2020)^64^ Zeiler *et al *(2023)^65^
	Anxiety or depression	Settipani *et al *(2013)^70^	Ozyurt *et al *(2016)^71^
Other	Eating disorder (any)	–	Duclos *et al* (2023)^48^ §
	Externalising	–	Aggarwal *et al* (2018)^47^ ¶
	Other diagnoses	–	–
Parenting stress/strain/burden			
PSI	Anxiety or depression	–	Farley *et al* (2023)^56^ Lebowitz *et al* (2020)^72^ Tan *et al *(2005)^45^
	Externalising	Timmer *et al* (2019)^53^	Acri *et al *(2016)^73^ He *et al* (2020)^44^
Other	Externalising	–	Aggarwal *et al *(2018)^47^
	Other diagnoses	Carroll *et al *(2022)^57^	–
General stress			
DASS	Anxiety or depression	–	Poole *et al* (2018)^68^ Halldorsson *et al *(2018)^46^
	Eating disorder (any)	–	Wilksch (2023)^55^
Overall mental health			
Various measures	Anxiety or depression	Derisley *et al* (2005)^43^	Ozyurt *et al* (2016)^71^
	Eating disorder (any)	Truttmann *et al *(2020) (SCL–90)^64^	Truttman *et al* (2020) (GHQ12)^64^ Zeiler *et al *(2023)^65^
	Externalising	Costin *et al* (2004)^74^	–
	Other diagnoses	–	Algorta *et al* (2018)^41^

* In all cases, includes OCD.

† In all cases, includes oppositional defiance disorder and conduct disorder.

‡ Anxiety was moderate for mothers of children with social anxiety and mild for those fathers, while it was mild for mothers of children with other anxiety and normal for those fathers.

§ Anxiety was normal in the fathers in this study, but mild in the mothers and mild overall.

¶ Anxiety was mild for mothers in this study, but not clinical for fathers.

OCD, obsessive compulsive disorder.

#### Study design and participant characteristics

Overall, the studies that were RCTs typically reported parent’s outcomes above threshold, and therefore within the clinical range. The only exception is Timmer *et al*,^53^ who reported below threshold scores of parenting stress. This is unsurprising, as we extracted only baseline scores (scope of this review relates to well-being, rather than interventions), and it is likely that those with higher levels of distress would be more attracted to take part in intervention studies. No patterns of results were observed in relation to the location of the study or the age of the CYP.

#### Parents’ depression scores

Of the 20 studies reporting parental depression scores, 9 had mean scores above the measures’ clinical cut-off, across different CYP diagnoses.

Seven studies reported percentage of sample with a clinical level of depression. Two used structured clinical interviews: 18% of parents overall had depression^54^ and 12.9% of mothers but just 3.9% of fathers (OR 3.64, 95% CI 0.77 to 17.15, p=0.102) had current major depression.^42^ The self-report studies reported depression rates ranging from 4.7% of fathers and 6.8% of mothers (of CYP with depression or anxiety)^52^ to 88% of mothers and 56% of fathers (of CYP with various psychiatric diagnoses).^51^

#### Parents’ anxiety scores

All 10 studies were with parents of CYP with anxiety and/or depression, or eating disorders. Eight of 10 studies reported means above the clinical range.

Four studies reported the percentage with clinical levels of anxiety. One study used a structured clinical interview, reporting clinical anxiety in 26.7% of mothers and 14.7% of fathers of children with anxiety.^42^ The studies using self-report data found clinical levels of anxiety in 27% of parents of CYP with eating disorder,^55^ 57.2% where CYP had an anxiety disorder^56^ and 4.5% mothers and 0% fathers of CYP with depression or anxiety.^52^

#### Parents’ parenting stress scores

None of these studies were with parents of CYP with eating disorders. Six of eight studies revealed means in the clinical range.

Three studies used self-report data and reported a percentage of parents with clinical levels of parenting stress, reporting 44% for parents of CYP with externalising difficulties,^53^ 73% of parents of CYP with conduct disorder^47^ and 27% of parents of CYP with diagnoses of psychosis.^57^

#### Parents’ general stress scores

Three studies reported general stress, all finding clinical levels.

One study reported the percentage scores in range of clinical general stress: 34.3% of parents of CYP with an eating disorder.^55^

#### Parents’ overall mental health

For overall mental health, four of seven studies had scores in the clinical range.

One study with parents of CYP with depression or anxiety measured personality difficulties, finding 2.3% of mothers and fathers in the clinical range of somatic difficulties, 4.5% of mothers and zero fathers in clinical range for avoidant personality difficulties and none in clinical range for antisocial personality difficulties.

#### Other findings

Aggarwal *et al*^47^ measured work and social adjustment in parents of CYP with conduct disorder, reporting mean scores showing ‘severe impairment’ for mothers but ‘low impairment’ for fathers, with a significant difference. Wilksch *et al*^58^ also reported of parents of CYP with eating disorders, 70.5% reported clinical levels of difficulties with their physical health and 92.7% reported clinical levels of difficulties with their romantic relationships. This study also reported that parents required a mean of 70 days of leave in total to care for their child, although no data from a control group are provided for comparison, nor is the timeframe over which these days were taken specified.

#### Studies examining time and recovery

Two studies reported data from at least two time points. With parents of CYP with ODD in China, He *et al*^44^ used a three-wave, cross-lagged design and measured depression and parenting stress in parents at point one, 1 and 2 years later in the same cohort of parents, finding that parental depression predicted parenting stress (rather than the other way around). Importantly however they were unable to control for the fact that only 42.7% of CYP were diagnosed with ODD by the 3-year follow-up point. They also found that the associations between parental depression and the PSI measures of parent-child dysfunctional interaction were bi-directional. Importantly, this was the case for their parents of CYP with ODD and their control group of parents of CYP without mental health difficulties, suggesting the finding is not specific to parenting CYP with mental health difficulties.

Wilksch^55^ specifically examined scores of parents of CYP with a current eating disorder versus parents of CYP who had recovered. The ‘recovered’ group had significantly better scores on all measured dimensions: physical health overall, emotional health overall, romantic relationship ratings and DASS scores for depression, anxiety and general stress.

## Discussion

This review investigated levels of well-being/distress among parents of CYP with mental health difficulties, and in relation to control groups (parents of CYP without mental health difficulties) where reported. There is an overall picture of poor well-being. Pooled means found depression was not typically in the clinical range, however anxiety, parenting stress and general stress were. However, the pattern of scores for individual studies is mixed, with some reporting below clinical threshold, particularly for depression. The percentages of participants with scores in the clinical range were inconsistent among studies. This may relate to differences in study methodology. Indeed, given numerous different combinations of design factors, for example, study type, CYP diagnosis, location of study, age of CYP and measure used, it is challenging to detect any patterns without studies specifically designed to do this. The data do not allow for clear comparisons to be made in relation to parental well-being by CYP diagnosis. Meta-analyses showed a pattern of higher distress in parents of CYP with mental health problems compared with parents of CYP without these diagnoses, and for mothers as compared with fathers of CYP with mental health problems, however sensitivity analysis finds some effects are not robust.

Sensitivity analysis for the meta-analyses showed the difference between case-control for parental depression was dependent on the presence of a study in China with parents of CYP with ODD.^44^ The mother-father difference in anxiety was no longer detected when a study from France with parents of CYP with anorexia nervosa was removed.^48^ These findings are difficult to interpret, owing to the divergence between the studies in measures, sample sizes and CYP diagnosis. However, it may be that the CYP condition itself is relevant. The qualitative research has shown that ODD can be experienced as a personal attack for parents,^59^ potentially linking to higher parental depression. For eating disorders, mothers report more fear than fathers and are more likely to be involved in highly significant moments, such as meal preparation,^60^ potentially amplifying anxiety in mothers.

A previous review, not limited to confirmed diagnoses, reported parental mental illness between 16% and 79% of parents of children receiving treatment from mental health services.^9^ Focusing on parents of CYP who self-harm, a review found mental health difficulties between 67% and 86% of parents.^14^ Rates of mental health difficulties in parents of CYP with ADHD were around 17%.^18^ A high-quality study using structured clinical interview found 18% of parents of CYP with mental health difficulties had depression.^54^ This variation continues in our review. Here, the studies reporting percentage of respondents scoring within the clinical range reported between 3.9%–88% for depression, with highest observed percentages in parents of children with depression and 4.5%–57.2% for anxiety, with highest observed percentages in parents of children with anxiety. This may relate to the impact of parental mental health on CYP’s mental health.^6^ Overall, the prevalence of distress in parents of CYP with mental health difficulties, linked by parents to their CYP’s difficulties, remains unclear. Likewise, variables associated with greater distress, including CYP, parent and family characteristics, are not well understood.

We identified few case-control studies. In research with parents of CYP with ADHD, case-control studies have established that these parents have higher levels of distress, compared with controls. Parenting stress was linked to the severity of CYP’s ADHD symptoms,^19^ however this question has not been addressed for parents of children with other conditions. Indeed, ADHD-focused systematic reviews identified k=53^18^ and k=22 published studies,^19^ for this one condition. Our review, covering far more conditions, identified k=32, suggesting a dearth of attention to the well-being of parents of CYP with other mental health conditions.

Causality cannot be established from these studies. Taken together our findings suggest that parents of children with mental health difficulties had poorer mental health than those whose children did not have mental health difficulties. It is well established that parents with mental health difficulties are more likely to have children with mental health difficulties.^6^,^8^ We cannot conclude from the included studies if the parents’ reported mental health was a consequence of their child’s distress, or had contributed to their child’s difficulties. Although all included measures were of ‘current’ well-being, none of the identified studies sought to account for previous history of mental health difficulties in parents. There were no measures of distress specifically relating to their CYP’s difficulties.

Parents of CYP with mental health difficulties experience anxiety and stress (parenting and general), and many experience elevated depression. Irrespective of underlying cause, it is important to make appropriate support available to parents, be that peer-support, parent-training, social services support or psychological therapy. CAMHS clinicians should be mindful of parents’ well-being and offer signposting. This may include parents to adult mental health services, however there is a need to develop services and evidence-based interventions that address the distress linked to these parents’ specific experiences with their CYP’s mental health difficulties. Policies may include reference to family support,^61^ and should include the need to develop appropriate pathways to support parents themselves.

The review has strengths. We followed standard, reproducible methods. There are very few reviews focusing on the issue of parental well-being where CYP has a mental health difficulty. Developing from the only other review we are aware of that specifically addresses this issue,^9^ our review included only studies with parents of CYP with an established clinical diagnosis. We focused exclusively on studies that used validated measures and compared parents of CYP with mental health difficulties with those of CYP without mental health difficulties. This approach aimed to specifically address issues around links between parental well-being and CYP diagnoses.

Our review has limitations. The age range may have excluded studies for psychoses, which typically emerges in late adolescence or early adulthood.^2^ Only including articles in English may limit the representativeness of our results, thus future studies may need to investigate potential differences with African and South American countries. The focus on measures with established cut-offs or designs with control groups was essential; however, it excluded studies on knowledge, information needs, parenting satisfaction and parenting self-efficacy. A further review is required to examine predictors of parental psychological well-being. Authors were not contacted for missing information, such as detailed sample sizes, meaning one study was excluded from the meta-analyses.^46^ Meta-analysis was conducted with few studies, and publication bias was not assessed.^62^ The literature itself is limited by lack of studies with control groups (we identified only k=5 across all included CYP conditions), minimal consideration of confounding variables such as parents’ gender, linsufficient attention to parents’ previous mental health and a lack of measures specifically addressing the impact of their CYP’s distress on parents.

Areas for future research include sufficiently powered, well-designed, representative studies that examine mental health in these parents, and quantify distress linked directly to the CYP’s conditions. The small number of case-control studies calls for a need for further research, to allow interpretation of findings and to truly begin to understand the level of distress among parents of CYP with mental health difficulties. Further longitudinal research is also required, along with greater consideration of factors associated with worse well-being for parents, for example, CYP risk to self, parents’ age, ethnicity, socio-economic status and social support. There is a lack of measures specific to distress linked to the CYP’s condition. Work is required to disentangle the reciprocal impact of parent and child mental health. Further attention is needed, particularly outside of the Global North, and with better reporting and inclusion of participants from a range of ethnic backgrounds, and those in different parenting roles. Further work on the psychological variables underpinning parent distress is required to select/design appropriate support interventions. Parent training interventions, for example, may focus on improving parental self-efficacy; however, the relationship of this variable to parent self-compassion or rumination, for example, is unknown.

### Conclusions and clinical implications

In summary, this review finds a mixed pattern, with some evidence for poor psychological well-being in parents have a CYP with a mental health difficulty. The meta-analyses indicated greater depression and parenting stress in parents of CYP with these difficulties compared with controls. Within parents of CYP with a mental health difficulty, mothers appear to experience higher levels of depression and anxiety. Further studies are needed to better understand the impact of CYPs’ mental health on parents’ well-being, particularly through longitudinal, case-control studies. These studies should also explore variations in relation to the specific CYP diagnosis. It remains important to consider parents’ well-being within CAMHS provision, as evidence indicates that distress can be high. Assessing and offering interventions where needed is crucial.

## Supplementary material

10.1136/bmjment-2023-300971 online supplemental file 1

10.1136/bmjment-2023-300971 online supplemental file 2

10.1136/bmjment-2023-300971 online supplemental file 3

10.1136/bmjment-2023-300971 online supplemental file 4

10.1136/bmjment-2023-300971 online supplemental file 5

10.1136/bmjment-2023-300971 online supplemental file 6

## Data Availability

No data are available.
