# Supplementary material for: Psychological well-being and needs of parents and carers of children and young people with mental health difficulties: a quantitative systematic review with meta-analyses
Source: BMJ Ment Health. 2024 Aug 2;27(1):e300971. doi: 10.1136/bmjment-2023-300971 (PMC11298743; doi:10.1136/bmjment-2023-300971)
Supplement: online supplemental file 1 [file bmjment-27-1-s001.docx]

Supplementary Materials Table S1 Study Characteristics and main findings

| **First**  **Author** | **Year** | **Country** | **Study design** | **Control**  **Sample** | **CYP Age**  **in years** | **CYP mental**  **health difficulty** | **Sample**  **size** | **% fathers** | **Measurement**  **tool used** | **Main Findings including mean**  **(s.d.) where relevant** |
| --- | --- | --- | --- | --- | --- | --- | --- | --- | --- | --- |
| Acri**^40^** | 2016 | USA | Controlled  trial | No | 7 to 11 | ODD | 320 | 9.4% | PSI-SF | Mean 98.6 (22.49) |
| Aggarwal**^41^** | 2018 | India | Cross- sectional | No | Mean 15 | CD | 32 | unclear | PSS, HAMA, WSAS | PSS: mothers 62.63 (16.00),  fathers 52.75 (13.9), p=0.01, d=0.66.  HAMA: mothers 19.4 (7.29),  fathers 10.9 (5.79). p<0.001. d=1.29.  WSAS: mothers 21.59 (8.33),  fathers 13.78 (7.52), p<0.001, d=0.98. |
| Algorta**^42^** | 2018 | UK | Case-control | Yes | Mean 9.4  (s.d. 1.9) | Bipolar | 621  (case:14 9, | unclear | GBI, Parent Stress Survey | Means - GBI case 36.14 (30.57),  control 31.40 (27.27), p=0.07.  Parent Stress Survey: Case |

|  |  |  |  |  |  |  | control: 491) |  | (Sisson & Fristad, 2001) | 24.34 (14.10), control 18.31  (12.64), p<0.01. |
| --- | --- | --- | --- | --- | --- | --- | --- | --- | --- | --- |
| CONAlqaht  ani**^43^** | 2020 | Saudi  Arabia | Cross-  sectional | No | Up to 18 | Anxiety | 220 | 39.5% | MCS-12 | Mean 46.8 (8.9) |
| Carroll**^44^** | 2022 | USA | Correlational | No | 6 to 17 | Psychoses | 56 | 16% | ZBI | 27% above cut-off for stress.  Mean 38.7 (16.39) |
| Cooper**^45^** | 2006 | UK | Case-control | Yes | 6 to 16 | Anxiety | 215 | 53.7% | SCID | Major depression: case 12.9% |
|  |  |  |  |  |  |  | (case:13 | (case:60 |  | mothers, 3.9% fathers, control |
|  |  |  |  |  |  |  | 6, | %, |  | 2.2% mothers, 0% fathers. OR |
|  |  |  |  |  |  |  | control: | control: |  | 3.64 (95% CI 1.05-64.2). |
|  |  |  |  |  |  |  | 79) | 43.0%) |  | Any anxiety diagnosis: case |
|  |  |  |  |  |  |  |  |  |  | 68.2% mothers, 27.5% fathers, |
|  |  |  |  |  |  |  |  |  |  | control 26.7% mothers, 14.7% |
|  |  |  |  |  |  |  |  |  |  | fathers. OR 5.9 (95% CI 1.68- |
|  |  |  |  |  |  |  |  |  |  | 10.43) |
| Costin**^46^** | 2004 | Australia | Comparison  two | No | 4 to 16 | ODD | 66 | unclear | GHQ-28 | Mean 29.1 (18.80) |

|  |  |  | intervention  s |  |  |  |  |  |  |  |
| --- | --- | --- | --- | --- | --- | --- | --- | --- | --- | --- |
| Derisley**^47^** | 2005 | UK | Case-control | Yes | 11 to 18 | Anxiety and OCD | 118 (case  1: 28,  case2: 28  , control: 62 ) | unclear | BSI | OCD group 58.1 (10.18), clinical  anxiety group 57.17 (9.69),  control group 51.73 (9.41). OCD and clinical anxiety significantly higher than control (d=0.65 and d=0.57  respectively). |
|  | 2023 | France | Cross- sectional | No | 13 to 21 | Anorexia Nervosa | 135 | 40.7% | HADS  (anxiety), BDI- II | HADS Fathers= 6.94 (3.63);  mothers= 9.63 (4.79) p<0.0001, d=0.63.  BDI-II: Fathers 8.76 (6.02),  Mothers14.88 (9.71), p=0.08, d=0.76 |
| Duclos**^48^** |  |  |  |  |  |  |  |  |  |  |
| Farley**^49^** | 2023 | Australia | Pilot  intervention (pre-post) | No | 3 to 7 | Anxiety Disorders | 21 | 4.8% | PSI-SF, BSI | 57.2 % above cut-off for clinical anxiety |

|  |  |  |  |  |  |  |  |  |  | BSI mean 2.91 (1.99). PSI-SF  total 95.2 (17.36) |
| --- | --- | --- | --- | --- | --- | --- | --- | --- | --- | --- |
| Fields**^50^** | 2012 | USA | RCT | No | Mean 9.9 | Depressive or bipolar disorders | 165 | 6.1% | HAMD, PDI | Mean HAMD 8.1 (7.5).  PDI reveals rate 18% clinical level. |
| Gerkensme yer**^51^** | 2008 | USA | Cross sectional | No | 2 to 19 | Internalizing and externalizing child behaviour  problems | 155 | 1% | CES-D | Mean 20 (12.62) |
| Halldorsson  **52** | 2018 | UK | Cross-  sectional, | No | 7 to 12 | Anxiety (Social  anxiety = SA and | 647 | 42.1% | DASS | Depression means SA 8.39  (7.75), OA 6.06 (7.16); Anxiety |
|  |  |  | two CYP |  |  | other anxiety = |  |  |  | means SA 5.31 (6.15), OA 3.66 |
|  |  |  | diagnosis |  |  | OA) |  |  |  | (5.11); general stress means SA |
|  |  |  | groups |  |  |  |  |  |  | 13.19 (8.33), OA 11.12 (8.45). |
| Hamovitch**^1^**  **0** | 2019 | USA | Cross-  sectional | No | 7 to 10 | ODD | 213 | 38% | CES-D-SF | Mean 7.68 (5.64) |

| He**53** | 2020 | China | Case- control, longitudinal | Yes | 4 to 11 | ODD | 521 (case  256,  control 265) | unclear | PSI-SF, CES-D | PSI: Parent distress - Case 33.56 (7.41); control 31.70 (7.22)  p=0.0039, d=0.36. Parent-child  - Case 28.01 (7.65); control  24.76 (6.97), p=0.0001, d=0.66.  Difficult child – Case 33.90 (8.69); control 28.81 (8.32), p=0.0001, d=0.87.  CES-D: Case: 10.07 (7.61)  Control 8.00 (6.41). p=0.0008 , d=0.33 |
| --- | --- | --- | --- | --- | --- | --- | --- | --- | --- | --- |
| He**54** | 2021 | China | longitudinal | No | 6 to 13 | ODD | 370 | 41.9% (155/370  reported fathers,  28 of  sample | CES-D | Mean 13.30 (8.28) |

|  |  |  |  |  |  |  |  | did not report) |  |  |
| --- | --- | --- | --- | --- | --- | --- | --- | --- | --- | --- |
| Johnco**^11^** | 2021 | Australia | Cross-  sectional | No | Mean  11.2 | Depression and  anxiety | 531  families | 4.14% | DASS | Mean 2.33 (3.66) |
| Lebowitz**^55^** | 2020 | USA | Randomized  Noninferiorit y | No | 7 to 14 | Anxiety | 124  families | unclear | PSI | Mean 133.6 (20.6) |
| Lim**^56^** | 2021 | Korea | Cross- sectional | No | 5 to 7 | Internalizing and externalizing  problems | 1358 | 50% | K6 | Mean fathers 10.96 (3.94),  mothers 11.54 (4.35). p=0.0003, d=0.14 |
| Ozyurt**^57^** | 2016 | Turkey | RCT | No | 8 to 12 | Anxiety | 50 | unclear | GHQ-28, STAI | Mean STAI-State 36.08 (7.00),  STAI-Trait 43.23 (9.28), GHQ28  Total: 4.62 (5.39) |
| Poole**^58^** | 2018 | Australia | RCT | No | 12 to 17 | Depressive  disorder | 64  families | 16.6% | DASS-21 | Mean 6.47 (7.40) |

| Racey**^59^** | 2018 | UK | Mixed  method, feasibility | No | 14 to 18 | Depression | 29 | 0% | BDI-II | Mean8.6 (7.7) |
| --- | --- | --- | --- | --- | --- | --- | --- | --- | --- | --- |
| Schwarte**^60^** | 2017 | Germany | Cross-  sectional | No | 11 to 18 | Anorexia nervosa | 296 | 82.9% | BDI-II | Mean 6.15 (3.99) |
| Sengupta**^61^** | 2017 | India | Cross- sectional | No | Not reported | Psychiatric | 100 | 50% | HDRS | Above cut-off for mild depression 88% mothers, 56%  fathers. OR 5.76 (95% CI 2.08-  15.97), p=0.0008. |
| Settipani**^62^** | 2013 | USA | Cross- sectional | No | 7 to 14 | Anxiety | 111 | focused  only on mothers | STAI | STAI-State: 29.53 (10.18). (Trait  not measured) |
| Stewart**^63^** | 2019 | UK | Pilot | No | Mean  15.6 | Bulimia nervosa | study 1:  34 | unclear | HADS | Mean: 6.15 (3.99) |
| Sung**^64^** | 2019 | Singapor  e | Cross-  sectional | No | 6 to 19 | Depression/  anxiety | 58 | 32.8% | ASR | Above clinical level for anxiety  – 4.5% mothers, 0% fathers. |

| Tan**^65^** | 2005 | Malaysia | Case-control | Yes | 9 to 16 | Depression | 141  (case: 82  , control: 59) | 41.9%  (case:  42.7%,  control: 40.7%) | BDI (original), PSI | PSI: Parental distress  Mothers: Case 29.2 (9.0),  control 30.1 (5.4). Fathers: Case  26.7 (8.6), control 29.3 (7.4).  Case mothers vs fathers p=0.21, d=0.28.  Parent-child interactions Mothers: Case 31.5 (10.7),  control 29.4 (6.3). Fathers: case  31.0 (7.9), controls 30.8 (7.2)  Mothers vs fathers p=0.82, d=0.05  Difficult child  Mothers: Case 31.5 (10.7),  control 31.3 (6.1). Fathers: Case  34.4 (8.2), control 30.8 (6.9)..  Mothers vs fathers p=0.18, d=0.40 |
| --- | --- | --- | --- | --- | --- | --- | --- | --- | --- | --- |

| Mothers: Case | (n=46) | 99.2 |
| --- | --- | --- |
| (21.3), Control | (n=35) | 90.8 |
| (14.2) |  |  |
| Fathers: Case | (n=36) | 92.8 |
| (21.3), control | (n=24) | 90.8 |
| (17.2) |  |  |

Case: mothers compared fathers p=0.1807, d=0,30.

BDI: Case: Mothers n=46, 10.02 (8.52). Fathers n=36 7.33 (8.17). p=0.15, d=0.32. Control:

Mothers n=36 6.89 (5.35),

Fathers n=24 7.50 (7.52). Case mothers significantly worse than control mother scores, case fathers n.s. to control fathers.

| Timmer**^66^** | 2019 | USA | RCT | No | 1 to 10 | Externalizing behaviour | 59 | unclear | PSI | Mean Parent distress 56.2 (26.7) – normal range  Parent-child dysfunction 61.1  (26.7) – normal  Difficult Child 76.5 (25.1) –  normal |
| --- | --- | --- | --- | --- | --- | --- | --- | --- | --- | --- |
| Truttmann**^6^**  **7** | 2020 | Austria | Quasi- randomised feasibility  trial | No | Mean 14.7 | Anorexia Nervosa | 102 | 14%  (gender) | GHQ12, SCL- 90, BDI-II, STAI | Means GHQ-12 4.7 (3.47),  SCL-90 = 0.43 (0.39), BDI-II  10.86 (7.02), STAI state 47.71  (10.66), STAI trait 41.49 (9.41) |
| Wilksch**^68^** | 2023 | Australia | Repeated  measures | No | <18 | Eating disorder | 372 | unclear | DASS‐21 | 34.3% above clinical cut-off.  Mean 16.41 (10.30) |
| Zeiler**^69^** | 2023 | Austria | Cross- sectional | No | 10 to 23 | anorexia nervosa | 248  (across 3 cohorts) | 59%-69%  across 3 cohorts | GHQ12, BDI-II, STAI | Means STAI state 45.4 (11.83),  STAI trait 40.4 (10.37), BDI-II  11.1 (8.01), GHQ12: 4.8 (3.63) |

Abbreviations: ASR – Adult Self Report; BDI – Beck Depression Inventory; BDI-II – Beck Depression Inventory version 2; BSI – Beck Symptoms Inventory; CES-D: Centre for Epidemiological Studies Depression Scale ; CES-D-SF: Centre for epidemiological Studies Depression short form; DASS: Depression Anxiety Stress Scale; DASS-21: Depression Anxiety Stress Scale 21 items; GBI: ; GHQ28: General Health Questionnaire 28 items;

GHQ12: General Health Questionnaire 12 items; HADS: Hospital Anxiety and Depression Scale ;HAMA: Hamilton Anxiety Rating Scale; HAMD: Hamilton Depression Rating Scale; HDRS: Hamilton Depression Ratin Scale; K6: Kessler Psychological Distress Scale; MCS-12:Mental health component scale of the 12-item Short Form ; PDI: Psychiatric Diagnostic Interview ;PSI: Parenting Stress Index; PSI-SF: Parenting Stress Index Short Form; PSS: Parental Stress Scale; SCID: Structured Clinical Interview for DSM Disorder ; SCL-90: Symptom Checklist 90; STAI: State Trait Anxiety Inventory ; WSAS: the Work and Social Adjustment Scale; ZBI: Zarit Burden Inventory
