# Supplementary material for: Psychological well-being and needs of parents and carers of children and young people with mental health difficulties: a quantitative systematic review with meta-analyses
Source: BMJ Ment Health. 2024 Aug 2;27(1):e300971. doi: 10.1136/bmjment-2023-300971 (PMC11298743; doi:10.1136/bmjment-2023-300971)
Supplement: online supplemental file 2 [file bmjment-27-1-s002.docx]

Table S2: Full searches for Research question 1

Medline

| S1 | TI ((parent or parents or parental or mother or father or care*giver or guardian* or carer*  or paternal or maternal) ) OR AB ( ( parent or parents or parental or mother or father or care*giver or guardian* or carer* or paternal or maternal ) ) OR MM ("Parents+") |
| --- | --- |
| S2 | TI ( (children or adolescent* or adolescence or youth* or child or teenager* or pediatric* or paediatric* or kid* or teen* or young person or young people or boy* or girl* or juvenile* ) ) OR AB ( ( children or adolescent* or adolescence or youth* or child or  teenager* or pediatric* or paediatric* or kid* or teen* or young person or young people or boy* or girl* or juvenile* ) ) OR MH ("Child+") OR MM ("Adolescent") |
| S3 | ( TI ( ( attention deficit disorder* or “attention deficit hyperactive disorder* “or “ADHD”) OR AB (attention deficit disorder* or “attention deficit hyperactive disorder* “or “ADHD”) OR MH (“Attention Deficit Disorder with Hyperactivity”) OR ( TI ( (Eating disorder* or anorexia or bulimia or eating problem*) ) OR AB ( (Eating disorder* or anorexia or bulimia or eating problem*) OR MH(“Feeding and Eating Disorders+”) OR ( TI ( (Emerging personality disorder* or emerging personality problem*) ) OR AB ( (Emerging personality disorder* or emerging personality problem*) ) OR MH (“Personality Disorder+”) ) OR ( TI ( (Externalising disorder* or externalising problem* or externalizing disorder* or externalizing problem*) OR AB (Externalising disorder* or externalising problem* or externalizing disorder* or externalizing problem*) OR TX (“Externalising disorder”) ) OR ( TI ( (Oppositional defiant disorder* or oppositional defiant problem*) ) OR AB ( (Oppositional defiant disorder* or oppositional defiant problem*) OR (MH“Attention Deficit and Disruptive Behavior Disorders+”) ) OR ( TI ( (Psychos* or psychotic disorder* or psychotic problem*) ) OR AB ( (Psychos* or psychotic disorder* or psychotic problem*) ) OR MH (“Psychotic Disorders+) ) OR ( TI ( ( Anxiety or depression or depressive or “obsessive compulsive disorder” or “OCD” or phobia or phobic or mood disorder or anxiety disorder or panic disorder or agoraphobia or internalising problem* or internalising problem* or internalizing problem* or internalizing disorder* ) ) OR AB ( ( Anxiety or depression or depressive or “obsessive compulsive disorder” or “OCD” or phobia or phobic or mood disorder or anxiety disorder or panic disorder or agoraphobia or internalising problem* or internalising problem* or internalizing problem* or internalizing disorder* ) OR (MH "Depressive Disorder") OR (MH "Depressive Disorder, Major") OR (MH "Depressive Disorder, Treatment-  Resistant") OR (MH "Dysthymic Disorder") OR (MM "Anxiety Disorders+") ) |
| S4 | TI (“Information need*” or “knowledge need*” or need* or support or experience*or impact or wellbeing or concern* or want or perspective* or belief* or attitude*or prefer* or anxiety or anxious or depressed or depression or strain or stress or burden or “parent satisfaction” or “family relationship” or “parent* self-efficacy”) OR AB (“Information need*” or “knowledge need*” or need* or support or experience*or impact or wellbeing or concern* or want or perspective* or belief* or attitude*or prefer* or anxiety or anxious or depressed or depression or strain or stress or burden or “parent satisfaction” or “family  relationship” or “parent* self-efficacy”)) |
| S5 | S1 N8 S4 |
| S6 | S2 N8 S3 |
| S7 | S5 AND S6 (English date limitation applied) |

PsycInfo, EMBASE, AMED, CINAHL

| S1 | (parent or parents or parental or mother or father or care*giver or guardian* or carer* or paternal or maternal).ab. or (parent or parents or parental or mother or father or care*giver  or guardian* or carer* or paternal or maternal).ti. or parents.kw. |
| --- | --- |

| S2 | (children or adolescent* or adolescence or youth* or child or teenager* or pediatric* or paediatric* or kid* or teen* or young person or young people or boy* or girl* or juvenile*).ab. or (children or adolescent* or adolescence or youth* or child or teenager* or pediatric* or paediatric* or kid* or teen* or young person or young people or boy* or  girl* or juvenile*).ti. or (child or adolescent).kw. |
| --- | --- |
| S3 | (attention deficit disorder* or attention deficit hyperactive disorder* or ADHD or Eating disorder* or anorexia or bulimia or eating problem* or Emerging personality disorder or emerging personality problem* or Externalising disorder* or externalising problem* or externalizing disorder* or externalizing problem* or Oppositional defiant disorder* or oppositional defiant problem* or Psychos* or psychotic disorder* or psychotic problem* or Anxiety or depression or depressive or obsessive compulsive disorder or OCD or phobia or phobic or mood disorder or anxiety disorder or panic disorder or agoraphobia or internalising problem* or internalising problem* or internalizing problem* or internalizing disorder*).ab. or (attention deficit disorder* or attention deficit hyperactive disorder* or ADHD or Eating disorder* or anorexia or bulimia or eating problem* or Emerging personality disorder or emerging personality problem* or Externalising disorder* or externalising problem* or externalizing disorder* or externalizing problem* or Oppositional defiant disorder* or oppositional defiant problem* or Psychos* or psychotic disorder* or psychotic problem* or Anxiety or depression or depressive or obsessive compulsive disorder or OCD or phobia or phobic or mood disorder or anxiety disorder or panic disorder or agoraphobia or internalising problem* or internalising  problem* or internalizing problem* or internalizing disorder*). |
| S4 | (information need* or knowledge need* or need* or support or experience*or impact or wellbeing or concern* or want or perspective* or belief* or attitude* or prefer* or anxiety or anxious or depressed or depression or strain or stress or burden or parent satisfaction or family relationship or parent* self-efficacy).ab. or (information need* or knowledge need* or need* or support or experience*or impact or wellbeing or concern* or want or perspective* or belief* or attitude* or prefer* or anxiety or anxious or depressed or  depression or strain or stress or burden or parent satisfaction or family relationship or parent* self-efficacy).ti. |
| S5 | S1 adj8 s4 |
| S6 | S2 adj8 s5 |
| S7 | S5 and s6 |

Web of Science – Complete Core Collection

| #1 | TS=(parent OR parents OR parental OR mother OR father OR care*giver OR guardian* OR carer* OR paternal OR maternal) |
| --- | --- |
| #2 | TS=(children OR adolescent* OR adolescence OR youth* OR child OR teenager* OR pediatric* OR paediatric* OR kid* OR teen* OR 'young person' OR 'young people' OR boy* OR girl* OR juvenile*) |
| #3 | TS=('attention deficit disorder*' OR 'attention deficit hyperactive disorder*' OR adhd OR 'attention deficit disorder with hyperactivity' OR 'Eating disorder* or anorexia or bulimia' OR 'eating problem*' OR 'feeding and eating disorders' OR 'emerging personality disorder*' OR 'emerging personality problem*' OR 'personality disorder' OR 'externalising disorder*' OR 'externalising problem*' OR 'externalizing disorder*' OR 'externalizing problem*' OR 'oppositional defiant disorder*' OR 'oppositional defiant problem*' OR 'attention deficit and disruptive behavior disorders' OR psychos* OR 'psychotic disorder*' OR 'psychotic problem*' OR anxiety OR depression OR depressive OR 'obsessive compulsive disorder' OR 'ocd' OR phobia OR phobic OR 'mood disorder' OR 'anxiety disorder' OR 'panic disorder' OR agoraphobia OR 'internalising problem*'  OR 'internalising disorder*' OR 'internalizing problem*' OR 'internalizing disorder*) |

| #4 | TS=(“Information need*” or “knowledge need*” or need* or support or experience*or impact or wellbeing or concern* or want or perspective* or belief* or attitude*or prefer*  or anxiety or anxious or depressed or depression or strain or stress or burden or “parent satisfaction” or “family relationship” or “parent* self-efficacy” ) |
| --- | --- |
| #5 | TS=((parent OR parents OR parental OR mother OR father OR care*giver OR guardian* OR carer* OR paternal OR maternal) NEAR/8 (“Information need*” or “knowledge need*” or support or experience* OR impact* or wellbeing or concern* or want or perspective* or belief* or attitude* or prefer* or anxiety or anxious or depressed or  depression or strain or stress or burden or “parent satisfaction” or “family relationship” or “parent* self-efficacy”)) |
| #6 | TS=(("attention deficit disorder" or "attention deficit hyperactive disorder" or adhd or "attention deficit disorder with hyperactivity" or "eating disorder" or "eating problem" or "feeding and eating disorder" or "emerging personality disorder" or "emerging personality problem" or "personality disorder" or "externalising disorder" or "externalising problem" or "oppositional defiant disorder" or "oppositional defiant problem" or "attention deficit and disruptive behavior disorders" or psychos* or "psychotic" or anxiety or depression or depressive or anxious or "obsessive compulsive disorder" or OCD or phobia or phobic or "mood disorder" or "anxiety disorder" or "panic disorder" or agoraphobia or "internalising problem" or "internalising disorder" or "internalizing problem" or "internalizing disorder" or "externalizing problem" or "externalizing disorder") NEAR/8 (children or adolescent* or adolescence or youth* or child or teenager* or pediatric* or paediatric* or kid* or teen* or "young person" or  "young people" or boy* or girl* or juvenile*)) |
| #7 | #5 AND #6 |
| #8 | #7 English |

Cochrane Library

| #1 | parent OR parents OR parental OR mother OR father OR care*giver OR guardian* OR carer* OR paternal OR maternal |
| --- | --- |
| #2 | children OR adolescent* OR adolescence OR youth* OR child OR teenager* OR pediatric* OR paediatric* OR kid* OR teen* OR 'young person' OR 'young people' OR  boy* OR girl* OR juvenile* |
| #3 | 'attention deficit disorder*' OR 'attention deficit hyperactive disorder*' OR adhd OR 'attention deficit disorder with hyperactivity' OR 'Eating disorder* or anorexia or bulimia' OR 'eating problem*' OR 'feeding and eating disorders' OR 'emerging personality disorder*' OR 'emerging personality problem*' OR 'personality disorder' OR 'externalising disorder*' OR 'externalising problem*' OR 'externalizing disorder*' OR 'externalizing problem*' OR 'oppositional defiant disorder*' OR 'oppositional defiant problem*' OR 'attention deficit and disruptive behavior disorders' OR psychos* OR 'psychotic disorder*' OR 'psychotic problem*' OR anxiety OR depression OR depressive OR 'obsessive compulsive disorder' OR 'ocd' OR phobia OR phobic OR 'mood disorder' OR 'anxiety disorder' OR 'panic disorder' OR agoraphobia OR 'internalising problem*'  OR 'internalising disorder*' OR 'internalizing problem*' OR 'internalizing disorder* |
| #4 | “Information need*” or “knowledge need*” or need* or support or experience*or impact or wellbeing or concern* or want or perspective* or belief* or attitude*or prefer* or  anxiety or anxious or depressed or depression or strain or stress or burden or “parent satisfaction” or “family relationship” or “parent* self-efficacy” |
| #5 | #1 and #2 and #3 and #4 |

WHO International Clinical Trials Registry Platform; Social Policy and Practice; Applied Social Sciences Index and Abstracts; and Open Grey

| #1 | parent OR parents OR parental OR mother OR father OR caregiver OR guardian* OR carer* OR paternal OR maternal |
| --- | --- |

| #2 | children OR adolescent* OR adolescence OR youth* OR child OR teenager* OR  pediatric* OR paediatric* OR kid* OR teen* OR 'young person' OR 'young people' OR boy* OR girl* OR juvenile* |
| --- | --- |
| #3 | 'attention deficit disorder*' OR 'attention deficit hyperactive disorder*' OR adhd OR 'attention deficit disorder with hyperactivity' OR 'Eating disorder* or anorexia or bulimia' OR 'eating problem*' OR 'feeding and eating disorders' OR 'emerging personality disorder*' OR 'emerging personality problem*' OR 'personality disorder' OR 'externalising disorder*' OR 'externalising problem*' OR 'externalizing disorder*' OR 'externalizing problem*' OR 'oppositional defiant disorder*' OR 'oppositional defiant problem*' OR 'attention deficit and disruptive behavior disorders' OR psychos* OR 'psychotic disorder*' OR 'psychotic problem*' OR anxiety OR depression OR depressive OR 'obsessive compulsive disorder' OR 'ocd' OR phobia OR phobic OR 'mood disorder' OR 'anxiety disorder' OR 'panic disorder' OR agoraphobia OR 'internalising problem*'  OR 'internalising disorder*' OR 'internalizing problem*' OR 'internalizing disorder* |
| #4 | “Information need*” or “knowledge need*” or need* or support or experience*or impact or wellbeing or concern* or want or perspective* or belief* or attitude*or prefer* or anxiety or anxious or depressed or depression or strain or stress or burden or “parent  satisfaction” or “family relationship” or “parent* self-efficacy” |
| #5 | #1 and #2 and #3 and #4 |
