# Supplementary material for: Psychological well-being and needs of parents and carers of children and young people with mental health difficulties: a quantitative systematic review with meta-analyses
Source: BMJ Ment Health. 2024 Aug 2;27(1):e300971. doi: 10.1136/bmjment-2023-300971 (PMC11298743; doi:10.1136/bmjment-2023-300971)
Supplement: online supplemental file 3 [file bmjment-27-1-s003.docx]

Table S3: Cut-off points to indicate normal, elevated, or clinical ranges of outcome measures used in included studies

| **Measure**  **Abbreviati on** | **Measure Name** | **Variable measured** | **Score Range** | **Cut-off point** | **Reference** |
| --- | --- | --- | --- | --- | --- |
| ASR | Adult Self-Report from the Achenbach System of Empirically Based Assessments | Anxiety, depression, and personality problems – somatic,  avoidant and antisocial. | 0-240 for total score | The thresholds for clinical and subclinical scores for the ABCL and ASR correspond to the 97th and 93rd percentiles. | Achenbach T.M., Rescorla L.A. Manual for the ASEBA Adult Forms & Profiles.  University of Vermont, Research Center for Children, Youth, &  Families; Burlington, VT,  USA: 2003 |
| BDI | Beck Depression Inventory (original 1961 measure) | Depression | 1 to 42 | 1-10:Normal 11-16:Mild  17-20 :Borderline clinical depression 21-30: Moderate  31-40 :Severe  40+: Extreme  The cut-off also used in the Turkish version, seen in Karacan, F. A., Yilmaz, S., & Kara, T. (2022). The Mental Health and Marital Adjustment of Mothers of Children with Attention Deficit  Hyperactivity Disorder. 60(3), pp. 220-227. | Beck At, Ward Ch, Mendelson M, Mock J, Erbaugh J. An inventory for measuring depression. Arch Gen Psychiatry 1961;4:561-  71. |
| BDI-II | BDI-II | Depression | 0 to 63 | 0-13 – minimal  14-19 – mild  20-28 – moderate  29 – 63 - severe | Beck, A. T., Steer, R. A., Ball, R., & Ranieri, W.  (1996). Comparison of Beck Depression Inventories -IA and -II in psychiatric outpatients. Journal of Personality Assessment, 67, 588–  597.  10.1207/s15327752jpa  6703. |

|  |  |  |  |  | | |  |
| --- | --- | --- | --- | --- | --- | --- | --- |
| BSI | Brief Symptom Inventory | Distress | Each subscale (T- score): 0 to  100 | **T[two scales] or GSI (Global severity index) t-scores:**  < 60: “no distress”  ≥ 60 and < 63: “mild distress”  ≥ 63 and < 70: “remarkable distress”  ≥ 70: “severe distress” | | | (Derogatis &  Melisaratos, 1983)  (Derogatis, 1993)  (Helga et al., 2021) |
|  |  |  |  | **PSDI (Positive symptom distress index) t-scores:**  < 49: “no distress”  ≥ 49 and < 60: “mild distress”  ≥ 60 and < 70: “remarkable distress”  ≥ 70: “severe distress” | | |  |
|  |  |  |  | **PST (Positive symptom total) t-scores:**  < 47: “no distress”  ≥ 47 and < 57: “mild distress”  ≥ 57 and < 69: “remarkable distress”  ≥ 69: “severe distress” | | |  |
| CES-D | Centre for Epidemiological Studies Depression Scale | Depression | 0 to 60 | 16+: Clinically significant depressive symptoms | | | (Hann D. et al., 1999) (Radloff, 1977) |
| DASS-42 | Depression Anxiety Stress | Depression, | Each | Depression: | Anxiety | Stress | (Lovibond & Lovibond, |
|  | Scale | anxiety, stress | subscale: 0 | 0-9: Normal | 0-7: Normal | 0-14: Normal | 1995) |
|  |  |  | to 42 | 10-13: Mild | 8-9: Mild | 15-18: Mild |  |
|  |  |  |  | 14-20: | 10-14: | 19-25: Moderate |  |
|  |  |  |  | Moderate | Moderate | 26-33: Severe |  |
|  |  |  |  | 21-27: Severe | 15-19: Severe | 34+ :Extremely Severe |  |
|  |  |  |  | + 28: | 20+: Extremely |  |  |
|  |  |  |  | Extremely | Severe |  |  |
|  |  |  |  | severe |  |  |  |

| DASS-21 | Depression Anxiety Stress | Depression, | Each | Depression: | Anxiety | Stress | (Henry JD & JR, 2005) |
| --- | --- | --- | --- | --- | --- | --- | --- |
|  | Scale – 21 items (Short | anxiety, stress | subscale: 0 | 0-4: Normal | 0-3: Normal | 0-7 Normal |  |
|  | version) |  | to 21 | 5-6: Mild | 4-5: Mild | 8-9 Mild |  |
|  |  |  |  | 7-10: | 6-7: Moderate | 10-12 Moderate |  |
|  |  |  |  | Moderate | 8-9: Severe | 13-16: Severe |  |
|  |  |  |  | 11-13: Severe | 10+: Extremely | 17+: Extremely Severe |  |
|  |  |  |  | + 14: | Severe |  |  |
|  |  |  |  | Extremely |  |  |  |
|  |  |  |  | severe |  |  |  |
| GBI | General Behavior Inventory (Depue et al.,  1982) |  |  | Depression – 22+  Hypomania – 13+ | | | (RL et al., 2002) |
| GHQ-12 | GHQ (short version 12 items) | Psychological disorder | 0 to 36 | **Cut-off threshold to identify “caseness” (ratio between experiment group scores and control group scores)**  < 2/3= 0.666: case of psychological disorder | | | (Anjara et al., 2020; Goldberg D. P. et al., 1998) |
|  |  |  |  | **Raw scores:**  Women:  <3 Non-cases Men  <3 Non-cases | | |  |
| GHQ-28 | GHQ (short version 28 items) | Psychological disorder | 0 to 84 | **Total scores:**  - 23: Non-psychiatric  + 24: Psychiatric | | | (Hjelle Ellen G. et al., 2019; Willmott Sasi A. et al., 2004)  (Pisula et al., 2019) |
|  |  |  |  | **Threshold for subscales:**  Low: 1/2 Mid:2/3  High :3/4 - 6/7 | | |  |
|  |  |  |  | Alternatively - “sten scores” 6 or more indicative of clinical difficulties. | | |  |
| HADS | Hospital and Anxiety and  Depression Scale | Anxiety and  depression | 0 to 21 | 0-7: Normal  8-10: Mild | | | (A.S Zigmond & Snaith,  1983) |

|  |  |  |  | 11-14 Moderate  15-21 Severe |  | | |
| --- | --- | --- | --- | --- | --- | --- | --- |
| HAMA / HARS | The Hamilton Anxiety Scale | Anxiety | 0 to 56 | <17: Mild  18-24: Mild to moderate  25-30: Moderate to severe | (W Maier et al., 1988) | | |
| HAMD / HDRS | The Hamilton Depression Scale | Depression | 0 to 29 | 0-7: Normal  8-13: Mild  14-18: Moderate  19-22: Severe  >23: Very severe | (E Frank et al., 1991) | | |
| HRSD-24 / HAMD24 | Hamilton Depression Scale  - 24 | Depression | 0 to 72 | < 8: no depression  8–19: mild depression  20–34: moderate depression  ≥ 35: severe depression | (LI et al., 2020) | | |
| K6 | Kessler 6 | Depression | 0 to 24 | 5 +: Moderate  13 +: Clinical | (Ronald C. Kessler et al., 2003) | | |
| MCS-12 | Mental health component  of the SF-12 | Mental  wellbeing | 0 to 100 | < 42: Clinical depression | (Ware et al., 1995) | | |
| PSI-SF (36  item versions) | Short version of the PSI | Stress | 36 to 180[^1^](#_bookmark0) | **Using percentile scores**  Parental distress: Typical 15-80; high stress 81-89; clinically significant stress 90-100  Parent-Child Dysfunctional interaction: Typical 5-80; high 81-84; clinically significant 85-100  Difficult child: 15-80; 81-89; 90-100  Total stress: 15-80; 81-89; 90-100  **Raw scores (clinical cutoff)**  Parental distress: > 33  Parent-Child Dysfunctional interaction: > 27 Difficult child: > 33  Total stress: > 90 | (Abidin, R. R., 1990)  (Dardas & Ahmad, 2014) | | |
| PSI4-SF | Parenting Stress Index Short Form version 4 | Stress | T-scores: 20  to 100 | T-scores:  < 62 within normal limits   - 66 clinically significant |  | https://www.tricare- west.com/content/dam  /hnfs/tw/prov/resource |  |

1 Range of scores is taken from sum of the 3subscales

f

Commented [SW1]: Website with information on scoring and results interpreation

|  |  |  |  |  |  | s/pdf/ACD%20PSI%20Pr  ovider%20education.pd |  |
| --- | --- | --- | --- | --- | --- | --- | --- |
|  |  |  |  |  |  |  | |
| PSS | Parental Stress Scale | Stress | 0 to 40 | Men cut-off: 24.0  Women cut-off: 25.6  Aggarwal paper has “severity of stress is rated as: Mild – 18 to 42; Moderate– 43 to 66; Severe – 67 to 90” with the scale going  from 18-90 – see [A Comparative Study of Stress, Anxiety & Work](https://journals.sagepub.com/doi/epdf/10.1177/0973134220180402) [Impairment in Parents of Children with Conduct Disorder](https://journals.sagepub.com/doi/epdf/10.1177/0973134220180402)  [(sagepub.com)](https://journals.sagepub.com/doi/epdf/10.1177/0973134220180402) | (Judy O. Berry & Jones, 1995) | | |
| SCL-90-R | Symptom Check-List-90-R (SCL-90-R) | Overall mental health | Mean of  item scores 0-4. | German version used in included study.  Cut-off for Global scale 0.5 indicates clinical difficulties | Schmitz, N., N.  Hartkamp, J. Kiuse, G.  H. Franke, G. Reister, &  W. Tress. (2000). The Symptom Check-List-90- R (SCL-90-R): A German  Validation Study. *Quality of Life Research*, *9*(2), 185–193.  [http://www.jstor.org/st](http://www.jstor.org/stable/4036992) [able/4036992](http://www.jstor.org/stable/4036992) | | |
| STAI | State-trait anxiety inventory | Anxiety (syndrome and trait) | 20 to 80 | Trait scale:   - 40 high anxiety traits State scale: - 40 high anxiety symptoms | (Addolorato et al., 1999) | | |
| WSAS | The Work and Social Adjustment Scale | Impairment in functioning | 0 to 40 | 0-9: Low impairment  10-19: Moderate impairment  20-40 Severe impairment | (James C Mundt et al., 2002) | | |
| ZBI | Zarit Burden Inventory | Burden | **22 item:**  0 to 88  **Short:** | **Revised version:**  0-21: No to mild burden  21-40: Mild to moderate Burden  41-60: Moderate to severe burden | (S. H Zarit et al., 1980) (Naser Al-Balushi et al., 2019) | | |

|  |  |  | 0 to 48  **Screen:**  0 to 16 | 61+: Severe burden  **Short version:**  0-10: No to mild burden  10-20: Mild to moderate burden 20+: High burden  **Screen version:**  8+: High burden |  |
| --- | --- | --- | --- | --- | --- |

A.S Zigmond , & Snaith, R. P. (1983). The hospital anxiety and depression scale. *67*, 361-370. <https://pubmed.ncbi.nlm.nih.gov/6880820/>

Addolorato, G., Ancona, C., Capristo, E., Graziosetto, R., Di Rienzo, L., Maurizi, M., & Gasbarrini, G. (1999). State and trait anxiety in women affected by allergic and vasomotor rhinitis. *Journal of psychosomatic research*, *46*(3), 283-289. <https://doi.org/10.1016/S0022-3999(98)00109-3>

Anjara, S. G., Bonetto, C., Van Bortel, T., & Brayne, C. (2020). Using the GHQ-12 to screen for mental health problems among primary care patients: psychometrics and practical considerations [OriginalPaper]. *International Journal of Mental Health Systems*, *14*(1), 1-13. https://doi.org/doi:10.1186/s13033-020-00397-0

Beck, A. T., Epstein, N., Brown, G., Steer,, A., R., & (1988). An Inventory for Measuring Clinical Anxiety: Psychometric Properties. *56*, 893-897.

Dardas, L. A., & Ahmad, M. M. (2014). Psychometric properties of the Parenting Stress Index with parents of children with autistic disorder. *Journal of Intellectual disability Research*, *58*(6). <https://doi.org/10.1111/jir.12053>

Depue, R. A., Slater, J. F., Wolfstetter-Kausch, H. K., Goplerud, D., & Eric Farr, D. (1982). A behavioral paradigm for identifying persons at risk for bipolar depressive disorder: A conceptual framework and five validation studies. *Journal of Abnormal Psychology*, *90*(5). <https://doi.org/10.1037/0021-843X.90.5.381>

Derogatis, L. R. (1993). *BSI Brief Symptom Inventory. Administration, Scoring, and Procedures Manual* (4th ed. ed.). Derogatis, L. R., & Melisaratos, N. (1983). The Brief Symptom Inventory: An introductory report. (13(3)), 595–605.

Diener, E. Emmons, R. A., Randy J Larsem, & Sharon Griffin. (1985). The Satisfaction With Life Scale. *49*, 71-75. Retrieved 2, from

E Frank, R. F Prien, R. B Jarrett, M. B Keller, D. J Kupfer, P. W Lavori, . . . Weissman, M. M. (1991). Conceptualization and rationale for consensus definitions of terms in major depressive disorder: Remission, recovery, relapse, and recurrence. *48*, 851-855.

Goldberg D. P., Oldehinkel T., J., O., & (1998). Why GHQ threshold varies from one place to another. *28*, 915-921. Retrieved 7, from <https://pubmed.ncbi.nlm.nih.gov/9723146/>

Hann D., Winter K., P., J., & (1999). Measurement of depressive symptoms in cancer patients: Evaluation of the center for epidemiological studies depression scale (CES-D).

*46*, 437-443. Retrieved 5, from

Helga, F. G., Melanie, J.-F., Dieter, K., & Katja, P. (2021). Frontiers | A New Routine for Analyzing Brief Symptom Inventory Profiles in Chronic Pain Patients to Evaluate Psychological Comorbidity. https://doi.org/doi:10.3389/fpsyg.2021.692545

Henry JD, & JR, C. (2005). The short-form version of the Depression Anxiety Stress Scales (DASS-21): construct validity and normative data in a large non-clinical sample. *Br J Clin Psychol.*, 227-239. [https://doi.org/10.1348/014466505X29657.](https://doi.org/10.1348/014466505X29657)

Hjelle Ellen G., Bragstad Line Kildal, Zucknick Manuela, Kirkevold Marit, Thommessen Bente, & Unni, S. (2019). The General Health Questionnaire-28 (GHQ-28) as an outcome measurement in a randomized controlled trial in a Norwegian stroke population. *7*. Retrieved 3, from <https://pubmed.ncbi.nlm.nih.gov/30902115/>

James C Mundt, Isaac M Marks, M. Katherine Shear, & Greist, J. H. (2002). The Work and Social Adjustment Scale: a simple measure of impairment in functioning. *180*, 461-

464. <https://pubmed.ncbi.nlm.nih.gov/11983645/>

Janssen Bas, & Agota, S. (2014). Population norms for the EQ-5D. 19-30. Retrieved 1, from <https://link.springer.com/chapter/10.1007/978-94-007-7596-1_3> Judy O. Berry, & Jones, W. H. (1995). The parental stress scale: Initial psychometric evidence. *12*, 463-472.

Kurt Kroenke, Robert L. Spitzer, & Williams, J. B. W. (2001). The PHQ-9: validity of a brief depression severity measure. *16*, 606-613. <https://pubmed.ncbi.nlm.nih.gov/11556941/>

Levine, S. Z. (2013). Evaluating the seven-item Center for Epidemiologic Studies depression scale short-form: a longitudinal U.S. community study. *48*, 1519-1526. Retrieved 9, from <https://pubmed.ncbi.nlm.nih.gov/23299927/>

LI, J., OAKLEY, L. D., BROWN, R. L., LI, Y., & LUO, Y. (2020). Properties of the Early Symptom Measurement of Post-Stroke D... : Journal of Nursing Research. *Journal of Nursing Research*, *28*(4). <https://doi.org/10.1097/jnr.0000000000000380>

Linn, M. (1985). A Global Assessment of Recent Stress (GARS) Scale. . *Int J Psychiatry Med*. <https://doi.org/> 10.2190/xp8n-rp1w-ye2b-9q7v Lovibond, S. H., & Lovibond, P. F. (1995). *Depression Anxiety and Stress Scales (DASS-42)*. https://doi.org/https://doi.org/10.1037/t39835-000

Naser Al-Balushi, Mohammed Al-Alawi, Muna Al Shekaili, Manal Al-Balushi, Hassan Mirza, Salim Al-Huseini, . . . Al-Adawi, S. (2019). Predictors of Burden of Care Among Caregivers of Drug-Naive Children and Adolescents With ADHD: A Cross-Sectional Correlative Study From Muscat, Oman. *23*, 517-526. Retrieved 3, from <https://pubmed.ncbi.nlm.nih.gov/30371135/>

Neha Shah, Mizaya Cader, Bill Andrews, Rose McCabe, & Stewart-Brown, S. L. (2021). Short Warwick-Edinburgh Mental Well-being Scale (SWEMWBS): performance in a clinical sample in relation to PHQ-9 and GAD-7. *19*, 1-9. Retrieved 12, from <https://hqlo.biomedcentral.com/articles/10.1186/s12955-021-01882-x>

Ocansey Patience M.E., Kretchy Irene A., Aryeetey Genevieve C., Agyabeng Kofi, & Justice, N. (2021). Anxiety, depression, and stress in caregivers of children and adolescents with mental disorders in Ghana and implications for medication adherence. *55*, 173. Retrieved 9, from /pmc/articles/PMC9334939/

/pmc/articles/PMC9334939/?report=abstract <https://www.ncbi.nlm.nih.gov/pmc/articles/PMC9334939/>

Pisula, A., Bryńska, A., Wójtowicz, S., Srebnicki, T., & Wolańczyk, T. (2019). General health, sense of coherence and coping styles in parents participating in Workshops for Parents of Hyperactive Children. *Psychiatria polska*, *53*(2), 419-432. https://doi.org/doi:10.12740/PP/94382

Radloff, L. S. (1977). The CES-D Scale: A Self-Report Depression Scale for Research in the General Population. *1*, 385-401.

RL, F., EA, Y., CK, D., D, D.-B., R, P.-D., L, T., & JR, C. (2002). Clinical decision-making using the General Behavior Inventory in juvenile bipolarity. *Bipolar disorders*, *4*(1). <https://doi.org/10.1034/j.1399-5618.2002.40102.x>

Ronald C. Kessler, Peggy R. Barker, Lisa J Colpe, Joan F Epstein, Joseph C Gfroerer, Eva Howes Hiripi, . . . Zaslavsky, A. M. (2003). Screening for Serious Mental Illness in the General Population. *60*, 184-189. Retrieved 2, from <https://jamanetwork.com/journals/jamapsychiatry/fullarticle/207204>

S. H Zarit, K. E Reever, & Bach-Peterson, J. (1980). Relatives of the impaired elderly: Correlates of feelings of burden. *20*, 649-655.

Skapinakis, P. (2014). Spielberger State-Trait Anxiety Inventory. 6261-6264. <https://link.springer.com/referenceworkentry/10.1007/978-94-007-0753-5_2825>

Skevington, S. M., Lotfy, M., & O'Connell, K. A. (2004). The World Health Organization's WHOQOL-BREF quality of life assessment: Psychometric properties and results of the international field trial. A Report from the WHOQOL Group. *Quality of Life Research*, *13*(2), 299-310. https://doi.org/10.1023/B:QURE.0000018486.91360.00

Spitzer Robert L., Kroenke Kurt, Williams Janet B.W., & Bernd, L. (2006). A brief measure for assessing generalized anxiety disorder: the GAD-7. *166*, 1092-1097. Retrieved 5, from <https://pubmed.ncbi.nlm.nih.gov/16717171/>

Tennant, R., Hiller, L.,Fishwick, R., Platt, S., Joseph, S., Weich, S., Parkinson, J., Secker, J., Stewart-Brown, S. (2007). Warwick-Edinburgh Mental Well-Being Scale. <https://psycnet.apa.org/doiLanding?doi=10.1037%2Ft01628-000>

W Maier, R Buller , M Philipp, & Heuser, I. (1988). The Hamilton Anxiety Scale: reliability, validity and sensitivity to chan ge in anxiety and depressive disorders. *J Affect Disord*. <https://doi.org/10.1016/0165-0327(88)90072-9>

Ware, J., Kosinski, M., & Keller, S. D. (1995). How to score the SF-12 physical and mental health summary scales Boston. *MA: The Health Institute*, *2*. Ware Jr, J. (2000). SF-36 health survey update. *Spine*, *25*(24). <https://doi.org/10.1097/00007632-200012150-00008>

Willmott Sasi A., Boardman Jed A.P., Henshaw Carol A., & W., J. P. (2004). Understanding General Health Questionnaire (GHQ-28) score and its threshold. *39*, 613-617.

Retrieved 8, from <https://pubmed.ncbi.nlm.nih.gov/15300371/>
