# Supplementary material for: Psychological well-being and needs of parents and carers of children and young people with mental health difficulties: a quantitative systematic review with meta-analyses
Source: BMJ Ment Health. 2024 Aug 2;27(1):e300971. doi: 10.1136/bmjment-2023-300971 (PMC11298743; doi:10.1136/bmjment-2023-300971)
Supplement: online supplemental file 4 [file bmjment-27-1-s004.docx]

**Supplementary Materials 4: Approach to study quality appraisal and results**

The approach to study quality appraisal is set out below, with the criteria detailed in Table S4.1 and the appraisal results in Table S4.2.

The scale covers three areas: selection of participants; comparability of case-control groups (where relevant); and exposure to the issue of interest.^1^ Each area has items that are scored with a star if criteria are met. This tool is commonly used to appraise quality in observational studies, and has versions for case-control and cohort studies, sometimes used for cross-sectional designs.^2,^ ^3^ The scale is modifiable ^3^. Our review includes a range of study designs, including cross-sectional, case-control, and intervention studies. However, our core research question relates to establishing the levels of our variables in our population at baseline”, rather than evaluating any type of intervention or longitudinal changes. As such, based on the originals and a version developed for cross-sectional studies^4^, we made modifications to increase relevance to our review. Modifications were made to specify what constitutes being a representative sample of cases and clarification regarding ascertainment of exposure (here exposure to CYP with a mental health condition). The details of comparability were expanded to allow evaluation not only of case-control designs, but also for cross-sectional designs around whether important confounding variables were considered. Given that our interest is not in repeated measures, follow-up considerations were altered to consider statistical testing and data completion. The criteria are detailed below in Table S3.1 and related to 1) selection of participants as representative, appropriate control group where included, how exposure to CYP mental health difficulties was ascertained, and definition of any control group; 2) comparability both in terms of comparison to any control group but also exploring within a sample the relevance of important factors such as presence of fathers in the sample and ethnic diversity; and 3) outcome assessment, testing and reporting of missing data. If the study provided acceptable evidence of the criteria, it was scored “*” and allocated a point. Otherwise the study scored “U” for unacceptable or “N/A” where the criteria was not relevant to that study. The maximum points available is nine, as in the original tool.

**Table S4.1: Study quality appraisal criteria and scoring details**

| **Criteria** | **Acceptable - scored “*”** | **Unacceptable – scored “U”** |
| --- | --- | --- |
| **Selection: Representative of the (exposed) sample** | Truly or somewhat representative – either statistically representative or drawn from an appropriate pool, e.g., parents of CYP who are attending services. | Intervention sample – whereby the characteristics and distress in parents taking part in an intervention may be different to the general parent population (e.g., worse therefore feeling need to attend intervention).  Selected convenience sample No details given |
| **Selection: Selection of non-exposure cohort**  **N.B. N/A for designs other than case-control** | Drawn from same / similar community as the exposed cohort | Drawn from a different cohort (e.g., parents in control group from a different ethnic group). No description given (where a  case-control study). |
| **Selection:**  **Ascertainment of exposure** | Clinical records revealing diagnosis of CYP mental health condition.  And/or | Self-reported symptoms of  distress, not in relation to clinical diagnosis |

|  | Researchers used validated method (e.g., structured clinical interview, appropriate screening tool) to confirm diagnosis of CYP mental  health condition | No information provided |
| --- | --- | --- |
| Selection: Definition of controls | CYP has no clinical records of distress and is not involved in clinical services or seeking support  Self-report by parent and/or CYP (e.g., CYPs have completed a screening measure for the mental  health condition of interest). | Assumed no clinical diagnosis without recording any attempts to ascertain this  No information provided |
| **Comparability: Maximum two stars**  ***N.B. may N/A where study sample is highly focused on e.g., mothers of CYP with specific diagnosis*** | One star: Study controls for or analyses in relation to main factor: parents’ gender.  Additional star: Study controls for additional relevant factor (e.g., parents’ ethnicity). | Study does not control for any relevant factors. |
| **Outcome: Assessment of outcome**  **N.B. this is an eligibility for inclusion criterion** | Clear outcomes are reported using validated measure with cut-off, or with comparison to control group. | No validated measures used. No outcome reported. |
| ***Outcome: Statistical test*** | Appropriate statistical test is used to describe the level of the observed variable in the sample, presented with appropriate confidence  intervals / p- value. | Statistical test not appropriate. No statistical test described. |
| **Outcome: None responses / incomplete data** | Missing data were justified (e.g., reasons for none responses or reasons for participant withdrawal  provided). | Missing data was not justified or no clear statement about missing data presented. |

**References**

1. Wells G, Shea B, O’Connell D, Peterson J, Welch V, Losos M, et al. The Newcastle-Ottawa Scale (NOS) for assessing the quality if nonrandomized studies in meta-analyses. Accessed February 2021 from: <http://wwwohrica/programs/clinical_epidemiology/oxfordasp.2012>.
2. Luchini C, Stubbs B, Solmi M, Veronese N. Assessing the quality of studies in meta-analyses: Advantages and limitations of the Newcastle Ottawa Scale. World Journal of Meta-Analysis. 2017;5(4):80-4.
3. Ma L-L, Wang Y-Y, Yang Z-H, Huang D, Weng H, Zeng X-T. Methodological quality (risk of bias) assessment tools for primary and secondary medical studies: what are they and which is better? Military Medical Research. 2020;7:1-11.
4. Herzog R, Álvarez-Pasquin MJ, Díaz C, Del Barrio JL, Estrada JM, Gil Á. Are healthcare workers’ intentions to vaccinate related to their knowledge, beliefs and attitudes? a systematic review. BMC Public Health. 2013;13(1):154.

**Table S4.2. The results of the study quality appraisal**

| **Study First Author** | **Selection: Representative of the**  **(exposed)**  **sample** | **Selection: Selection of non-exposure cohort** | **Selection: Ascertainment of exposure** | **Selection: Definition of controls** | **Comparability:** | **Outcome: Assessment of outcome** | **Outcome: Statistical test** | **Outcome: None responses / incomplete**  **data** | **Total**  **/9** |
| --- | --- | --- | --- | --- | --- | --- | --- | --- | --- |
| Acri | * | * | * | NA | U | * | * | * | 6 |
| Aggarwal | * | NA | * | NA | * | * | * | NA | 5 |
| Algorta | * | * | * | U | U | * | * | NA | 5 |
| Alqahtani | * | NA | U | NA | ** | * | * | NA | 5 |
| Carroll | * | NA | * | NA | U | * | U | NA | 3 |
| Cooper | * | U | * | * | * | * | * | * | 7 |
| Costin | U | NA | * | NA | U | * | * | * | 4 |
| Derisley | * | U | * | NA | U | * | * | NA | 4 |
| Duclos | * | NA | * | NA | * | * | * | * | 6 |
| Farley | U | NA | * | NA | U | * | * | * | 4 |
| Fields | U | NA | * | NA | U | * | * | NA | 3 |
| Gerkensmeyer | U | NA | * | NA | ** | * | * | * | 6 |
| Halldorsson | * | NA | * | NA | * | * | * | * | 6 |
| Hamovitch | * | NA | U | NA | U | * | * | * | 4 |
| He, 2020 | U | U | * | * | ** | * | * | * | 7 |
| He, 2021 | U | NA | * | NA | U | * | * | NA | 3 |
| Johnco | * | NA | U | NA | U | * | * | * | 4 |
| Lebowitz | * | NA | * | NA | U | * | * | * | 5 |
| Lim | * | NA | U | NA | * | * | * | NA | 4 |
| Ozyurt | * | NA | * | NA | U | * | * | * | 5 |
| Poole | * | NA | * | NA | U | * | * | * | 5 |
| Racey | * | NA | * | NA | U | * | * | * | 5 |
| Schwarte | * | NA | * | NA | * | * | U | * | 5 |
| Sengupta | * | U | * | NA | * | * | * | U | 5 |
| Settipani | * | NA | * | NA | U | * | U | * | 4 |
| Stewart | * | NA | U | NA | U | * | * | * | 4 |
| Sung | * | NA | * | NA | ** | * | U | NA | 5 |
| Tan | * | * | * | U | * | * | * | NA | 6 |

| Timmer | U | NA | * | NA | U | * | U | * | 3 |
| --- | --- | --- | --- | --- | --- | --- | --- | --- | --- |
| Truttmann | * | NA | * | NA | U | * | * | * | 5 |
| Wilksch | * | NA | U | NA | U | * | * | U | 3 |
| Zeiler | * | NA | * | NA | U | * | * | U | 4 |
