# Supplementary material for: Psychological well-being and needs of parents and carers of children and young people with mental health difficulties: a quantitative systematic review with meta-analyses
Source: BMJ Ment Health. 2024 Aug 2;27(1):e300971. doi: 10.1136/bmjment-2023-300971 (PMC11298743; doi:10.1136/bmjment-2023-300971)

Supplementary Materials 5: Forest plots

Figure S5.1 Forest plots comparing case-control data for a) depression and b) parent stress


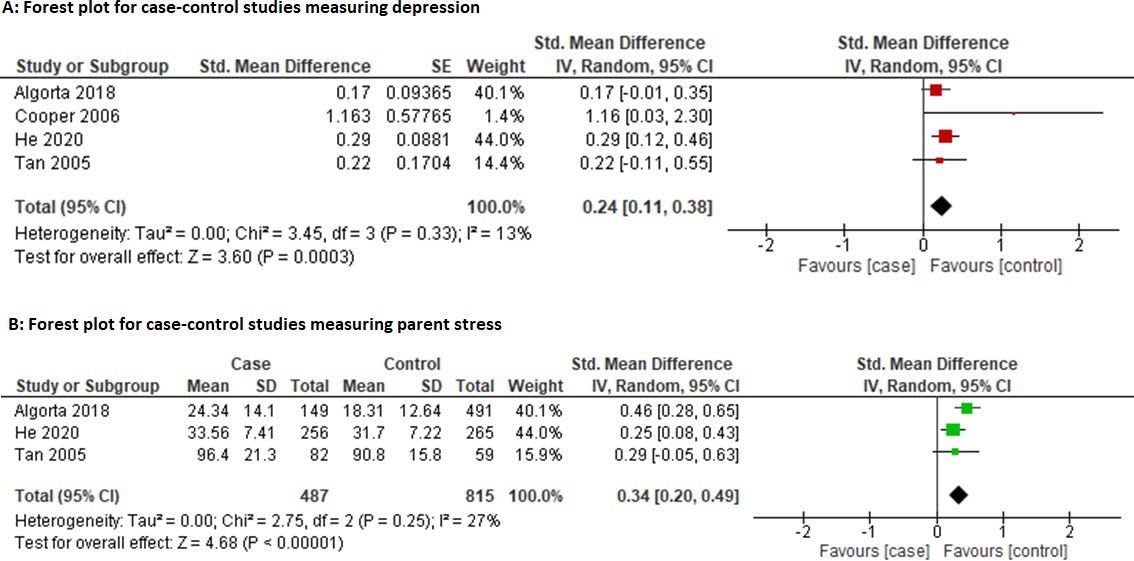


Figure S5.2 Forest plots comparing data from mothers and fathers for a) depression and b) anxiety


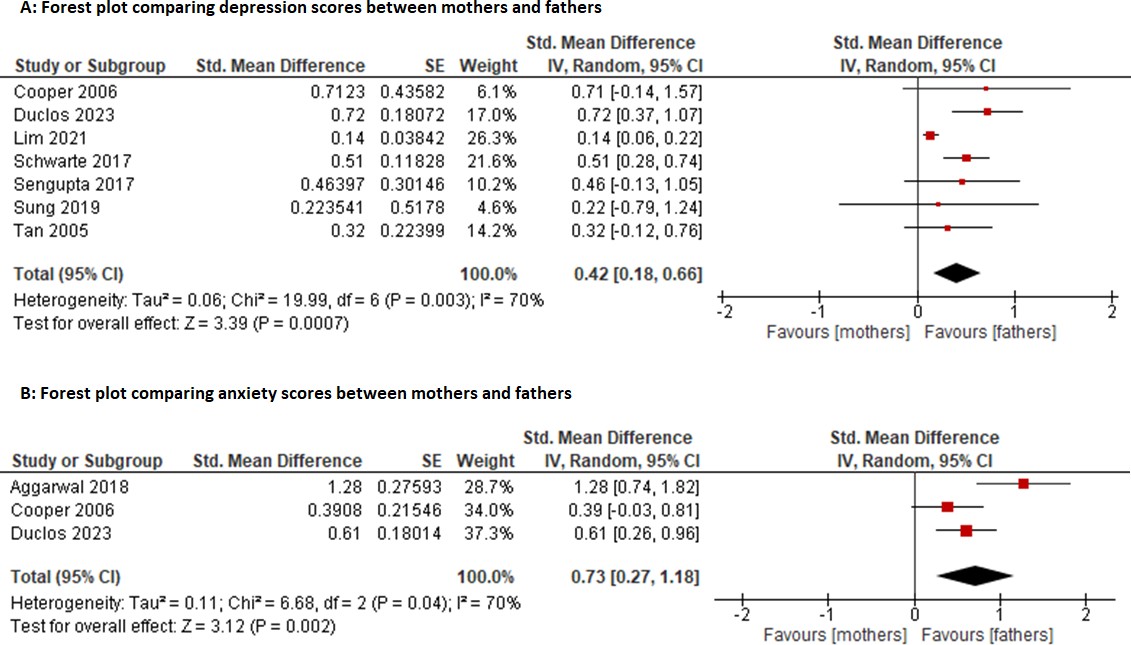

Supplement: online supplemental file 5 [file bmjment-27-1-s005.docx]
