# Supplementary material for: Psychological well-being and needs of parents and carers of children and young people with mental health difficulties: a quantitative systematic review with meta-analyses
Source: BMJ Ment Health. 2024 Aug 2;27(1):e300971. doi: 10.1136/bmjment-2023-300971 (PMC11298743; doi:10.1136/bmjment-2023-300971)
Supplement: online supplemental file 6 [file bmjment-27-1-s006.docx]

Supplementary Materials 6: Sensitivity Analysis

Sensitivity analysis was performed by removing each study in turn from each meta-analysis comparison. Table S6.1 provides the details of the impact of this on the report mean differences and 95% confidence intervals.

Table S6.1

| **Comparison** | **Outcome** | **Study deleted** | **Mean difference**  **(95% CI)** |
| --- | --- | --- | --- |
| Case-control | Depression | All in | 0.24 (0.11-0.38) |
|  |  | Algorta 2018 | 0.30 (0.10-0.50) |
|  |  | Cooper 2006 | 0.23 (0.11-0.35) |
|  |  | He 2020 | 0.23 (-0.01-0.47)* |
|  |  | Tan 2005 | 0.22 (0.07-0.4%) |
| Case-control | Parenting stress | All in | 0.34 (0.20-0.49) |
|  |  | Algorta 2018 | 0.26 (0.11-0.41) |
|  |  | He 2020 | 0.42 (0.26-0.59) |
|  |  | Tan 2005 | 0.36 (0.15-0.56) |
| Mothers-fathers | Depression | All in | 0.42 (0.18-0.66) |
|  |  | Cooper 2006 | 0.40 (0.15-0.65) |
|  |  | Duclos 2003 | 0.34 (0.12-0.56) |
|  |  | Lim 2021 | 0.53 (0.36-0.69) |
|  |  | Schwarte 2017 | 0.39 (0.12-0.67) |
|  |  | Sengupta 2017 | 0.41 (0.15-0.67) |
|  |  | Sung 2019 | 0.43 (0.17-0.68) |
|  |  | Tan 2005 | 0.44 (0.16-0.71) |
| Mothers-fathers | Anxiety | All in | 0.73 (0.17-1.18) |
|  |  | Aggarwal 2018 | 0.52 (0.25-0.79) |
|  |  | Cooper 2006 | 0.91 (0.26-1.57) |
|  |  | Duclos 2023 | 0.82 (-0.05-1.69)* |

*95% CI crosses zero and no longer statistically significant at p<0.05

For case-control depression, study characteristics are here, in Table S6.2

| **Study** | **n** | **CYP condition** | **Country** | **Measure** | **Significant**  **difference?** |
| --- | --- | --- | --- | --- | --- |
| Algorta 2018 | 612 | Bipolar | UK | GBI | No |
| Cooper 2006 | 215 | Anxiety | UK | SCID | Yes |
| He 2020 | 521 | ODD | China | CESD | Yes |
| Tan 2005 | 141 | Depression | Malaysia | BDI | No |

For mother-father anxiety, study characteristics are here, in Table S6.3

| **Study** | **n** | **CYP condition** | **Country** | **Measure** | **Significant**  **difference?** |
| --- | --- | --- | --- | --- | --- |
| Aggarwal 2018 | 32 | ODD | India | HAMA | Yes |
| Cooper 2006 | 215 | Anxiety | UK | SCID | No |
| Duclos 2023 | 135 | Anorexia | France | HADS | Yes |
